# Phylogenetic characteristics and molecular epidemiological analysis of novel enterovirus EV-B83 isolated from Tibet, China

**DOI:** 10.1038/s41598-020-63691-4

**Published:** 2020-04-20

**Authors:** Jinbo Xiao, Yong Zhang, Mei Hong, Zhenzhi Han, Man Zhang, Yang Song, Dongmei Yan, Shuangli Zhu, Wenbo Xu

**Affiliations:** 1 WHO WPRO Regional Polio Reference Laboratory, National Labortory for Poliomyelitis and National Health Commission Key Laboratory for Biosafety, National Institute for Viral Disease Control and Prevention, Chinese Center for Disease Control and Prevention, Beijing, People’s Republic of China; 2 Tibet Center for Disease Control and Prevention, Lhasa City, Tibet Autonomous Region, People’s Republic of China; 3 Center for Biosafety Mega-Science, Chinese Academy of Sciences, Beijing, 102206 People’s Republic of China

**Keywords:** Virology, Preventive medicine

## Abstract

Enterovirus B83 (EV-B83) is a new member of the enterovirus B group. Currently, there are only two full-length genomic sequences of EV-B83 in the GenBank database and few *VP1* region sequences. The aetiology and epidemiology of EV-B83 is unclear. 24 stool specimens were collected from twelve AFP patients and 298 stool specimens were collected from 298 healthy children in support of polio eradication activities in Tibet in 1999. Two polioviruses (isolated by L20B cell) and one non-polio enterovirus (isolated by RD cell) were isolated from AFP patients and nine polioviruses (isolated by L20B cell) and 90 non-polio enteroviruses (isolated by RD cell) were isolated from health children. Through molecular typing, we confirmed that the six of non-polio enteroviruses belong to EV-B83. The sequence similarity between the *VP1* region of the Tibet isolates and that of the EV-B83 prototype strain was 80%. The maximum-likelihood phylogenetic tree of the partial *VP1* region in EV-B83 demonstrated that EV-B83 formed four genotypes globally during the evolution process. The six Tibet EV-B83 strains formed the D genotype alone. Recombination analysis of Tibet EV-B83 showed that CV-B4, CV-A9, EV-B80, and EV-B106 may act as recombinant donors in multiple regions. The serum neutralization test showed that the antibody-positive rate was 58.8% and GMT was 1:19.70, which was higher than the previously reported results of EV-B106 and EV-B80. Temperature sensitivity test results showed that the six Tibet EV-B83 strains were temperature-insensitive with stronger virulence and potential infectivity, which was consistent with the results of the serum neutralization test. This study enriched the genome-wide sequence, epidemiological characteristics, and provided basic data for the follow-up study of EV-B83.

## Introduction

The genus *Enterovirus* of family *Picornaviridae* and order *Picornavirales* includes the species enterovirus (EV) A–L and rhinovirus (RV) A–C. The types that primarily cause human diseases are EV-A–D and RV-A–C^1^. Human enteroviruses include over 100 types, comprising poliovirus, coxsackievirus, echovirus, and some newly discovered enteroviruses. Human enterovirus can cause various infectious diseases, including acute flaccid paralysis (AFP); hand, foot, and mouth disease; acute aseptic meningitis; encephalitis; and others. Human enterovirus is a small, non-enveloped, single-stranded positive RNA virus of approximately 7500 nucleotides (nt), including a 5′-untranslated region (UTR), single long open reading frame (ORF), and *3*′*-UTR*^2,3^. The ORF translates into a polyprotein approximately 2200 amino acids long, which can be cleaved into three polyprotein precursors, *P1*, *P2*, and *P3*, and further divided into structural proteins *VP4*, *VP2*, *VP3*, and *VP1* and non-structural proteins *2A*, *2B*,* 2C*,* 3 A*, *3B*,* 3C*, and *3D*. Based on the *VP1* coding region sequence, human enteroviruses are classified into different types. This molecular typing method for type determination has gradually replaced the traditional neutralization test typing method^4–8^.

Currently, EV-B includes newly identified enteroviruses (types 69, 73–75, 77–88, 93, 97–98, 100–101, 106–107, 110–113), coxsackievirus A group (type 9), coxsackievirus B group (types 1–6), echovirus (types 1–7, 9, 11–21, 24–27, 29–33), and simian enterovirus SA5.

Enterovirus B83 (EV-B83) is a novel type within EV-B. The prototype strain (CA76-10392/USA/1976) of EV-B83 was isolated in the United States in 1976 and first reported in 2007. The GenBank database contains only two full-length genomic sequences of EV-B83: those of the EV-B83 prototype strain isolated in the United States^9^ and EV-B83 (*246/YN/CHN/08HC*) isolated in Yunnan, China^10^. Additionally, there are only 12 *VP1* sequences of EV-B83 that are over 600 nt in length in GenBank, and there are few studies and reports on EV-B83.

In this study, we identified six EV-B83 strains isolated from Tibet (strains 99245*/XZ/CHN/1999*, 99258*/XZ/CHN/1999*, 99267*/XZ/CHN/1999*, 99268*/XZ/CHN/1999*, 99269*/XZ/CHN/1999*, and 99275*/XZ/CHN/1999*, hereafter referred to as *99245*, 99258, *99267*, *99268*, *99269*, and *99275*). One Tibet EV-B83 strain (99258) was isolated from an AFP patient, indicating high virulence. Whether EV-B83 can cause more serious clinical diseases like some EV-B viruses is unclear^11–15^; therefore, further study of its epidemiology and other characteristics is necessary. In this paper, we will describe six Tibet EV-B83 strains from the following aspects: genome-wide sequence characteristics, phylogenetic relationships, recombination characteristics, epidemiological characteristics.

## Results

### Viral isolation and Molecular typing

Two polioviruses (isolated by L20B cell) and one non-polio enterovirus (isolated by RD cell) were isolated from AFP patients and nine polioviruses (isolated by L20B cell) and 90 non-polio enteroviruses (isolated by RD cell) were isolated from health children. The Online Enterovirus Genotyping Tool identifies the *VP1* region of each strain to show that six non-polio enteroviruses (*99245*, *99258*, *99267*, *99268*, *99269*, and *99275*) belong to EV-B83^16^. BLAST was used for sequence alignment, which showed that the *VP1* region of the six strains and the EV-B83 prototype strain (*CA76-10392/USA/1976*) exhibited 80% similarity in the GenBank.

Strains *99245* and *99258* were isolated from stool specimens of healthy children in Ngari Prefecture and an AFP patient in Linzhi City, respectively, in 1999. Strains *99267*, *99268*, *99269*, and *99275* were isolated from stool specimens of healthy children in Linzhi City in 1999.

### Full-length genome sequence analysis of six strains

The six Tibet EV-B83 strains were 7396 nt in length with a single 6552 nt ORF encoding a polypeptide of 2184 amino acids. The *5*′*-UTR* and *3*′*-UTR* comprised 744 and 100 nt, respectively. The base composition of all six EV-B83 strains was 28.2–28.3% A, 23.8–23.9% T, 23.5–23.6% C, and 24.3–24.4% G. We compared the genome-wide sequences of six EV-B83 strains with that of the EV-B83 prototype strain (*CA76-10392/USA/1976*) and found that the nucleotide insertion and deletion sites in the six EV-B83 strains were identical and located in the *5*′*-UTR* region, all of which had two nucleotide insertions at position 101, one at position 152, and three at position 722. There was one nucleotide deletion at position 129 and three at position 695. Pairwise comparisons of the nucleotide sequences and deduced amino acid sequences were conducted among the six Tibet EV-B83 strains, the prototype strain of all EV-B strains, and the Yunnan EV-B83 strain (*246/YN/CHN/08HC*) (Table 1). We also compared the nucleotide similarity between the *P1*, *P2*, and *P3* regions of the six EV-B83 strains and those of the prototype EV-B strain. In the *P1* region, the six EV-B83 strains had the highest nucleotide similarity with the Yunnan EV-B83 and EV-B83 prototype strains. However, in the *P2* and *P3* regions, the six Tibet strains had the highest nucleotide similarity with CV-B6 (GenBank accession number AF039205) and EV-B106 (GenBank accession number KF990476), respectively. These indicate that the six Tibet strains EV-B83 may show recombination with other EV-B strains in the *P2* and *P3* regions. The nucleotide and amino acid similarities among the full-length genomic sequences of six Tibet EV-B83 strains were 97.9–99.9% and 99.1–100.0%, respectively, while those between the six strains and the EV-B83 prototype strain were 81.0–81.1% and 96.4–96.6%, respectively.

**Table 1:** Pairwise comparisons of nucleotide sequences and deduced amino acid sequences were conducted among the six Tibet EV-B83 strains and the EV-B83 prototype strain (CA*76-10392/USA/1976*).

			*99245/XZ/CHN/1999*	*99258/XZ/CHN/1999*	*99267/XZ/CHN/1999*	*99268/XZ/CHN/1999*	*99269/XZ/CHN/1999*	*99275/XZ/CHN/1999*
*5*′*-UTR*		**nt (%)**	82.1	82.4	82.4	82.4	82.4	82.4
*P1*	*VP4*	**nt (%)**	76.8	77.7	77.7	77.7	77.7	77.7
		**aa (%)**	94.2	94.2	94.2	94.2	94.2	94.2
	*VP2*	**nt (%)**	80.6	81.3	81.0	81.0	81.0	81.2
		**aa (%)**	96.5	96.1	95.7	95.7	95.7	95.7
	*VP3*	**nt (%)**	78.0	78.0	78.0	77.9	78.0	78.0
		**aa (%)**	97.0	97.4	97.4	97.4	97.4	97.4
	*VP1*	**nt (%)**	79.4	79.8	79.3	79.6	79.6	79.6
		**aa (%)**	95.0	95.7	94.7	95.7	95.7	95.7
*P2*	*2A*	**nt (%)**	78.0	78.2	78.4	78.0	78.2	78.2
		**aa (%)**	97.3	97.3	97.3	97.3	97.3	97.3
	*2B*	**nt (%)**	81.8	81.8	81.4	81.8	81.8	81.4
		**aa (%)**	95.9	95.9	95.9	95.9	95.9	95.9
	*2 C*	**nt (%)**	81.0	81.3	81.1	81.2	81.2	81.1
		**aa (%)**	95.7	96.0	96.0	96.0	96.0	96.0
*P3*	*3A*	**nt (%)**	77.9	77.9	77.9	77.9	77.9	78.2
		**aa (%)**	96.6	96.6	96.6	96.6	96.6	96.6
	*3B*	**nt (%)**	83.3	80.3	80.3	80.3	80.3	80.3
		**aa (%)**	100.0	100.0	100.0	100.0	100.0	100.0
	*3C*	**nt (%)**	82.8	81.9	82.5	82.1	82.3	82.3
		**aa (%)**	98.9	98.9	98.9	98.9	98.9	98.9
	*3D*	**nt (%)**	83.2	82.7	82.8	82.7	82.6	82.7
		**aa (%)**	96.9	96.7	96.7	96.7	96.7	96.7
*3*′*-UTR*		**nt (%)**	91.2	92.2	92.2	92.2	92.2	92.2

### Phylogenetic analysis of six Tibet EV-B83 strains

**Figure 1:**
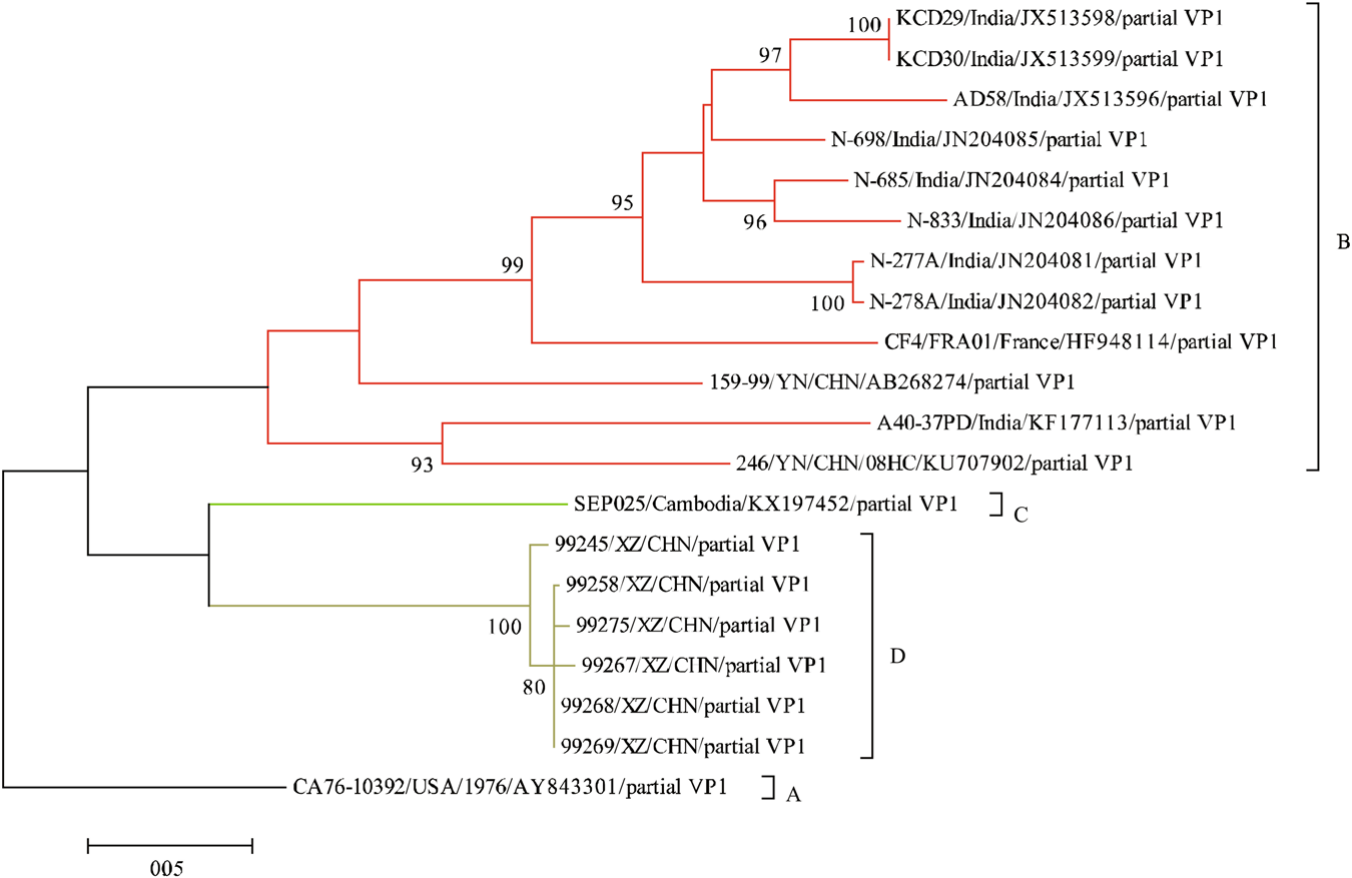
Maximum-likelihood phylogenetic tree based on a partial *VP1* comprising 633 nt (2528–3160 nt in the EV-B83 prototype strain). Different colours represent different genotypes. Numbers on nodes indicate the bootstrap support of the node (1000 bootstrap replicate percentage). Scale bars represent the replacement of each site per year.

The phylogenetic tree based on the partial *VP1* region (633 nt) (Fig. 1) showed that the *VP1* region greatly differed in genetic distance compared to other regions, and the selected EV-B83 sequences were further divided into genotypes. We named the prototype strain (CA76-10392/USA/1976) of EV-B83 isolated in the United States in 1976 as genotype A, and the rest were divided according to the time of isolation. EV-B83 isolated from Cambodia (GenBank accession number KX197452) was classified as genotype C, the six Tibet EV-B83 strains were classified as genotype D, and the remaining EV-B83 including Chinese Yunnan strains, Indian strains, and French strain composed genotype B. We verified genotype classification by analysing inter-group and intra-group distances and found that the average distance between the four groups was 21–25%, which met the criteria of 15–25% for defining genotype by nucleotide differences in enteroviruses^8,17^.

**Figure 2:**
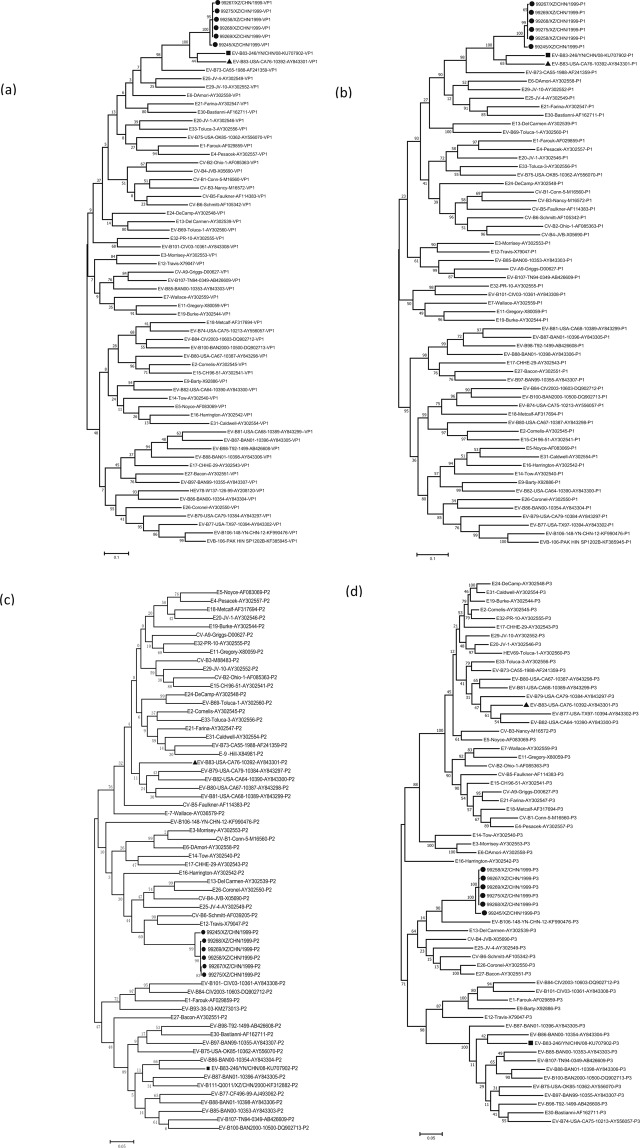
Maximum-likelihood phylogenetic trees based on *VP1*, *P1*, *P2*, and *P3* coding regions of the prototype sequence of all EV-B in the GenBank database and sequences of Yunnan EV-B83 and six Tibet EV-B83 strains. Numbers on nodes indicate the bootstrap support of the node (1000 bootstrap replicate percentage). Scale bars represent the replacement of each site per year. Coding sequences of (**a**) *VP1*, (**b**) *P1*, (**c**) *P2*, and (**d**) *P3* are shown.

Based on the sequences of all EV-B prototype strains in the GenBank database and those of the Yunnan EV-B83 strain and the six Tibet EV-B83 strains, we constructed phylogenetic trees based on *VP1*, *P1*, *P2*, and *P3* coding regions, respectively (Fig. 2). Both *VP1* and *P1* phylogenetic trees showed that the six Tibet EV-B83 strains were clustered with the EV-B83 prototype and Yunnan strains with a 100% bootstrap value, which confirmed previous molecular typing results. However, the phylogenetic trees constructed based on the *P2* and *P3* coding regions showed different results. In the *P2* region, the six Tibet EV-B83 strains were clustered with the CV-B6 prototype strain (GenBank accession number AF039205) and the E-12 prototype strain (GenBank accession number X79047). In the *P3* region, they were clustered with EV-B106 (GenBank accession number KF990476). This is consistent with previous results regarding nucleotide similarities. It is also suggested that six Tibet EV-B83 strains may have recombination in the *P2* and *P3* coding regions, leading to a large difference in the nucleotide sequence between them and the previously isolated EV-B83 strains in these regions.

### Analysis of recombination patterns of six strains

**Figure 3:**
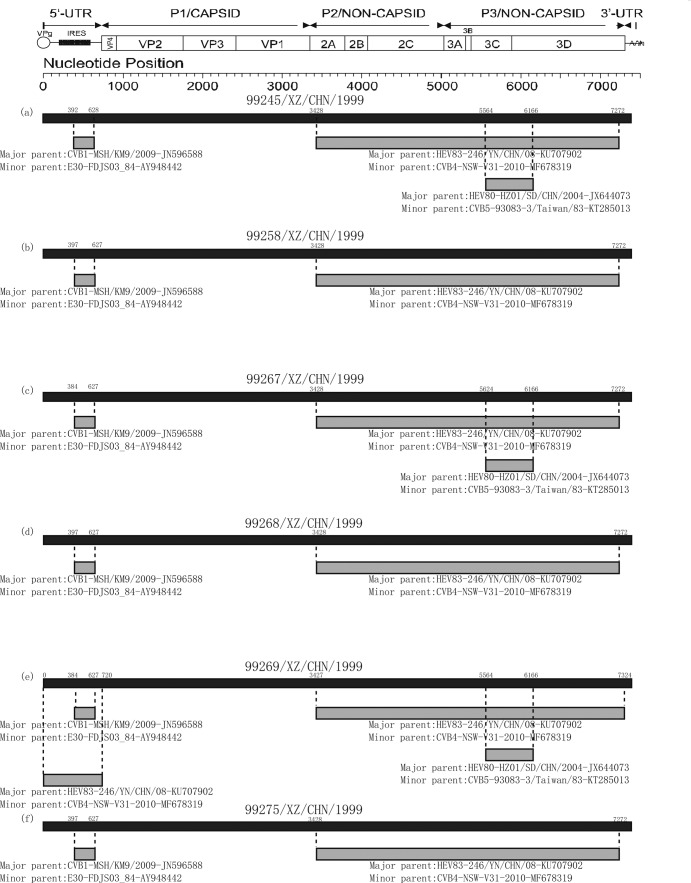
The genomic map of six Tibetan EV-B83 recombination events predicted by RDP4. The black band represents the full-length genome of six EV-B83; the number above indicates beginning and ending breakpoint positions. The grey band represents the genomic region where recombination events may occur; the number below indicates major and minor parents of the predicted recombination event. This figure is consistent with Table 2. (**a**) *99245/XZ/CHN/1999*; (**b**) *99258/XZ/CHN/1999*; (**c**) *99267/XZ/CHN/1999*; (**d**) *99268/XZ/CHN/1999*; (**e**) *99269/XZ/CHN/1999*; (**f**) *99275/XZ/CHN/1999*.

Sequence analyses of the six Tibet EV-B83 strains by RDP4 showed that recombination patterns in these strains were largely alike^18^. The breakpoint positions of three Tibet EV-B83 strains (*99258*, 99268, 99275) are mainly located in 397–627 and 3428–7272, covering part or all of the *5*′*-UTR* and *2A*–*3D* regions of the genome. The breakpoint positions of the other three Tibet strains (99245, 99267, 99269) are located in 392(384)–628(627), 3428(3427)–7272(7324), and 5564(5524)–6166, covering part or all of the *5*′*-UTR*, *2A*–*3D*, and *3*′*-UTR* genomic regions, respectively (Fig. 3). Although the recombination analysis results of the six Tibetan strains showed some differences at the breakpoint positions, considering the high sequence similarities, we still selected all primary and secondary potential parents (CV-B1, E-30, CV-B4, EV-B80, CV-B, CV-A, EV-B106) for recombination analysis using the SimPlot program (v3.5.1)^19^. The results of SimPlot and BootScan, the maximum-likelihood phylogenetic tree, and the high similarity of recombination sites of the CV-A9, CV-B4, and Tibet EV-B83 strains led to the determination that EV-B106, EV-B80 (GenBank accession number JX644073), partial CV-A9 in the GenBank, and some CV-B4 strains were recombined with the six Tibet EV-B83 strains. Because the recombination sites of the CV-A9, CV-B4, and Tibet EV-B83 strains were very similar, we used the SimPlot program to analyse the Tibet EV-B83 strains in two methods.

**Figure 4:**
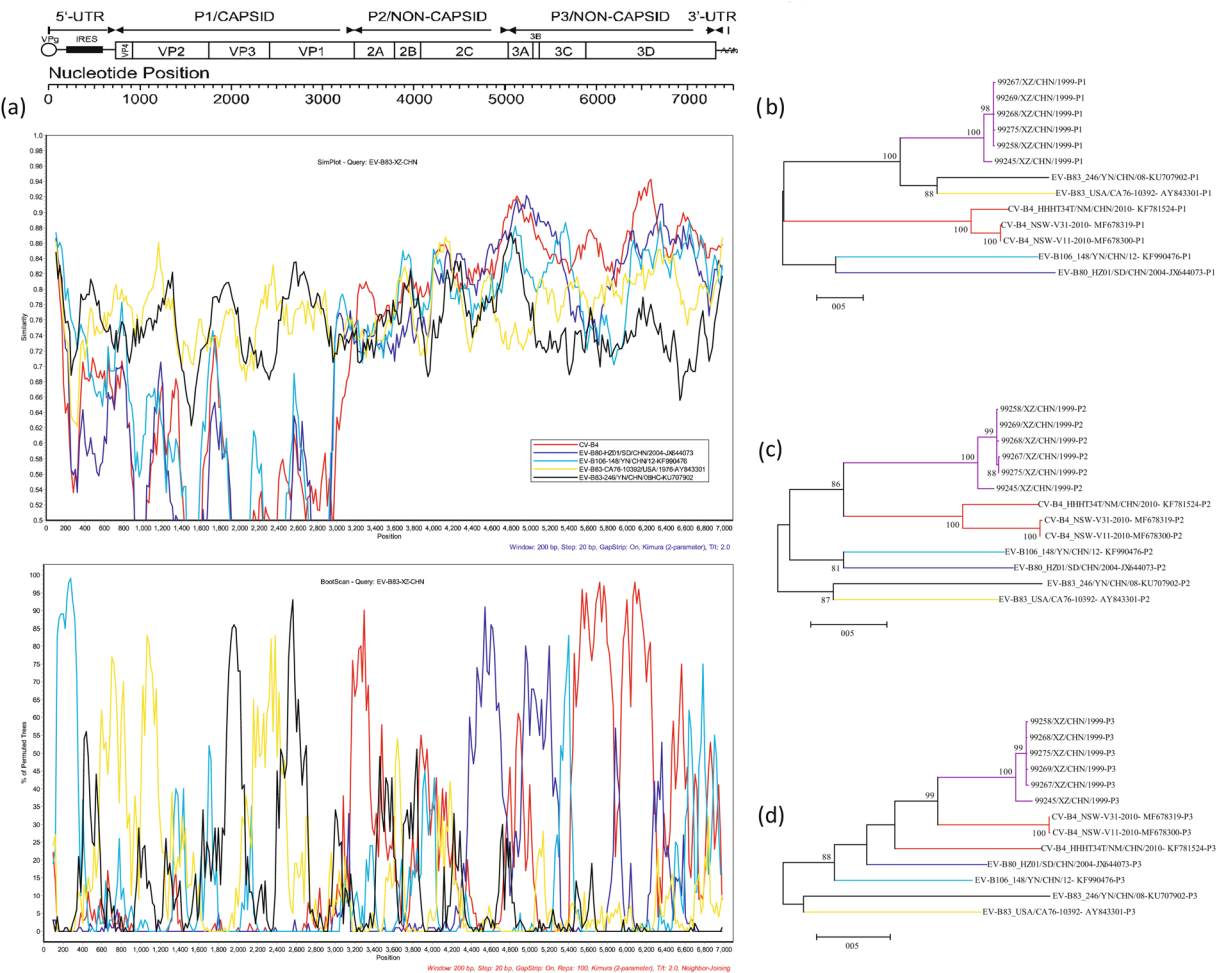
Recombination analyses of the four Tibet EV-B83 strains with CV-B4 strains. (**a**) EV-B83 Tibet group as the query sequence. Similarity and bootscanning analysis performed by Tibet EV-B83 group with EV-B83 prototype strain (*CA76-10392/USA/1976*), EV-B83 Yunnan strain (*246/YN/CHN/08*), EV-B80 (*HZ01/SD/CHN/2004*), EV-B106 (*148/YN/CHN/12*), and CV-B4 group. Maximum-likelihood phylogenetic trees were constructed based on the (**b**) *P1*, (**c**) *P2*, and (**d**) *P3* regions of the above sequences. Among these, purple and red branches in (**b**–**d**) represent the EV-B83 Tibet and CV-B4 groups, respectively. Each branch colour coincides with the colour of the similarity and bootscanning analysis results.

First, we performed recombinant analysis while excluding CV-A9 (Fig. 4). The BootScan results showed that when Tibet EV-B83 was used as the query sequence for analysis, there was a strong recombination signal in the *5*′*-UTR* and *VP4* partial regions between EV-B106 and the Tibet EV-B83 group. In the *P2* regions, the CV-B4, EV-B80, and Tibet EV-B83 groups showed a smaller range of recombination in *2B* and *2 C*, respectively. In the *P3* regions, the CV-B4 and Tibet EV-B83 groups had high recombination in the *3D* regions. This result had a high support value, which is consistent with the results of RDP4.

**Figure 5:**
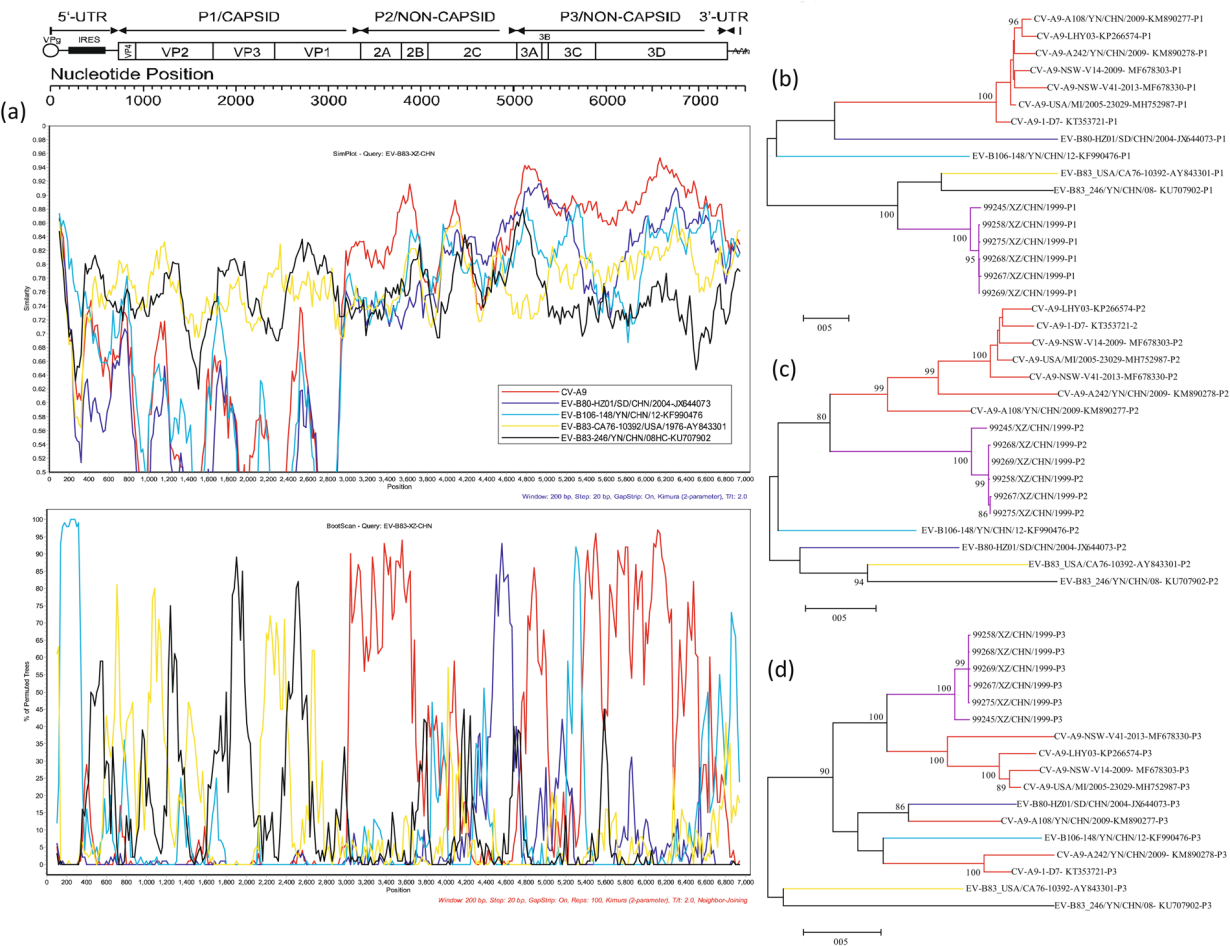
Recombination analyses of the four Tibet EV-B83 strains with CV-A9 strains. (**a**) EV-B83 Tibet group as the query sequence. Similarity and bootscanning analysis performed by Tibet EV-B83 group with EV-B83 prototype strain (*CA76-10392/USA/1976*), EV-B83 Yunnan strain (*246/YN/CHN/08*), EV-B80 (*HZ01/SD/CHN/2004*), EV-B106 (*148/YN/CHN/12*), and CV-A9 group. Maximum-likelihood phylogenetic trees were constructed based on the (**b**) *P1*, (**c**) *P2*, and (**d**) *P3* regions of the above sequences. Among these, purple and red branches in (**b**–**d**) represent the EV-B83 Tibet and CV-A9 groups, respectively. Each branch colour coincides with the colour of the similarity and bootscanning analysis results.

Second, recombination analysis was performed while excluding CV-B4 (Fig. 5). The BootScan results showed that EV-B106 and EV-B83 had a strong recombination signal in the *5*′*-UTR* and *VP4* regions when Tibet EV-B83 was used as the query sequence for analysis. In the *P2* regions, the EV-B80, EV-B106, and Tibet EV-B83 groups had a smaller range of recombination signals in *2C* and *3C*, which also verified the above recombination results. The CV-A9 and Tibet EV-B83 groups had a wide range of reorganization, which covered most regions of *2A–2C* and *3D* and small regions of *3B* and *3C*.

In both cases, the aggregation of the maximum-likelihood phylogenetic tree in the *P1*, *P2*, and *P3* regions of each sequence confirmed the results of our recombination analysis.

### Sero-epidemiology of EV-B83 in tibet

We randomly selected serum samples from 51 children in the Tibet (39 in Lhasa and 12 in Shigatse) for sero-epidemiology study. Serum control and cell control are all normal. The results showed that 30 of 51 serum samples were seropositive (>1:80) at a positive rate of 58.8%, and the GMT of the positive samples was 1:19.70. The composition ratios of neutralization antibody titters of <1:8, 1:8–1:64, and >1:64 were 41.2%, 56.9%, and 1.9%, respectively. Compared with other EV-B (such as EV-B106 and EV-B80) previously reported in China^20,21^, positive EV-B83 rates were higher among children of the same age. There were 19 positive samples in Lhasa with a 48.7% positive rate and GMT of 1:22.22. In Shigatse, there were 11 positive samples with a 91.7% positive rate and GMT of 1:16. Further analysis by the chi-square test (*χ*^2^ = 5.33, P < 0.05) showed a significant difference in the positive rate between the two regions.

### Temperature sensitivity test of six strains

**Figure 6:**
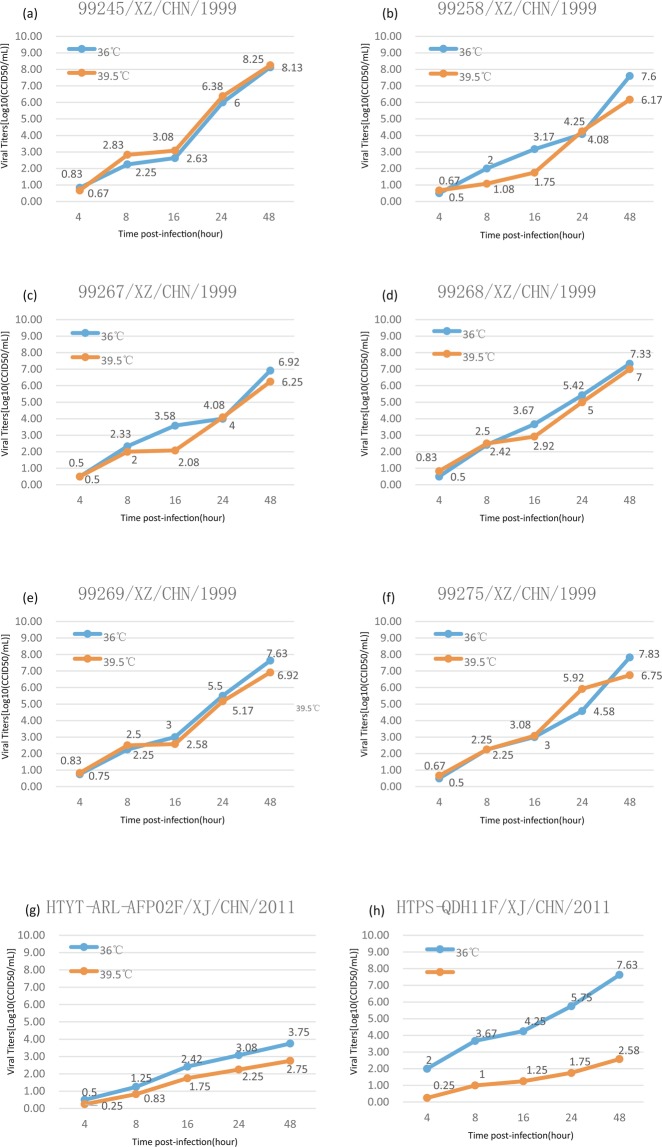
A titre-time line chart of six EV-B83 temperature sensitivity experiments. The blue and red lines represent line charts of the virus titre changing over time at 36 and 39.5 °C, respectively. Xinjiang EV-B106 strain (*HTPS-QDH11F/XJ/CHN/2011*) and Xinjiang EV-B85 strain (*HTYT-ARL-AFP02F/XJ/CHN/2011*) were used as temperature-sensitive and –insensitive controls, respectively. (**a**) *99245/XZ/CHN/1999*; (**b**) *99258/XZ/CHN/1999*; (**c**) *99267/XZ/CHN/1999*; (**d**) *99268/XZ/CHN/1999*; (**e**) *99269/XZ/CHN/1999*;(**f**) *99275/XZ/CHN/1999*; (**g**) strain *HTYT-ARL-AFP02F/XJ/CHN/2011*(EV-B85); (**h**)strain *HTPS-QDH11F/XJ/CHN/2011*(EV-B106).

After the six Tibet strains were cultured at 36 °C and 39.5 °C, the titre test showed that they were not temperature-sensitive strains (Fig. 6). Although their replication ability varied slightly at different temperatures, differences in the titre at 36 °C and 39.5 °C did not exceed two logarithms, and their logarithmic growth curves at the two temperatures were also highly similar. Unlike the previously reported temperature-sensitive EV-B106 and some temperature-sensitive EV-A90 strains, six Tibet EV-B83 strains is more temperature resistant.

## Discussion

The emergence of molecular typing technology has greatly changed the current situation and accelerated the discovery of novel enteroviruses, which have been increasingly reported^9,22–24^. Currently, there are only two EV-B83 full-length genomic sequences and twelve in the *VP1* region with lengths over 600 bp available in GenBank. Moreover, the epidemiology of EV-B83 remain unclear, so research regarding EV-B83 is valuable.

The full-length genomic sequences of six Tibet EV-B83 strains were highly similar. Comparison of these sequences with that of the EV-B83 prototype strain showed that their insertion and deletion sites were identical and all located in the *5*′*-UTR* region. Although the six EV-B83 strains came from different areas of Tibet, the high consistency in sequences indicates that they may share a common origin, and EV-B83 may have a relatively limited spread between the two places.

A phylogenetic tree constructed based on all available *VP1* regions (633 nt in length) of EV-B83 illustrated an interesting phenomenon: the six Tibet EV-B83 strains form an independent evolutionary branch and are far from the two EV-B83 strains isolated from Yunnan, China, indicating that EV-B83 has two different evolutionary branches in China^10,25^. We divided all EV-B83 into four genotypes, A, B, C, and D, based on nucleotide diversity in the *VP1* region. This division of genotypes clarifies and simplifies the evolutionary relationship of EV-B83.

The number of EV-B types is larger than that of EV-A, and the recombination between the genotypes is also very frequent^26–28^. Many articles have discussed the reorganization of different novel EV-B strains^20,21,29^. We know that recombination and nucleotide mutations promote the evolution of enteroviruses and new type emergence. Recombinant analysis is also crucial in epidemiological studies. We found that six Tibet EV-B83 strains and many previously discovered new enteroviruses (EV-B80, EV-B106, etc.) have reliable recombination in multiple genomic regions.

The serum neutralization test is greatly important for studying virus prevalence. The change in serum antibody is a dynamic process that can objectively evaluate the prevalence and spread of the virus^30–32^. According to serum neutralization test results for children in Lhasa and Shigatse, the seroprevalence and GMT of EV-B83 are higher than those of previously reported enteroviruses, such as EV-B106, EV-A90, and EV-B80^20,21,24^, indicating that EV-B83 is more widespread than some of previously discovered enteroviruses and may result in potential outbreaks and mass epidemics. The temperature sensitivity test is a good indicator of virulence and contagiousness^33^. The six Tibet EV-B83 strains showed resistance to temperature changes, indicating that they have high virulence and potential contagiousness. This is consistent with our previous suggestion that EV-B83 is more common than other EV (EV-B106, EV-A90).

In recent years, more and more novel enteroviruses have been detected; this is closely related to the high recombination frequency between enteroviruses. This high-frequency recombination promotes the evolution of enteroviruses, but also leads to the emergence of recombinant enteroviruses with unknown aetiology and epidemiology, such as EV-B83 reported in this study, which shows a high resistance to temperature and higher prevalence than previously reported novel enteroviruses (such as EV-B106 and EV-B80). We should strengthen the monitoring of these novel enteroviruses and use a variety of emerging technologies to improve the detection rate in specimens in order to identify those that may cause outbreaks as early as possible^34–37^.

By analysing six Tibet EV-B83 strains, this paper elucidated epidemiological characteristics of EV-B83 and enriched its full-length sequence in the GenBank database. These findings will aid in further research on EV-B83.

## Materials and Methods

### Sample collection

This study involved no human experimentation. National Labortory for Poliomyelitis in China CDC has established national laboratory network for detecting pathogens from AFP cases, their contactors, and healthy children in China. Stool samples of AFP patients and healthy children in Tibet served as the experimental specimens. The main purpose of the study is public health. This study was approved by the second session of the Ethics Review Committee of the National Institute for Viral Disease Control and Prevention (IVDC), Chinese Center for Disease Control and Prevention, all experimental protocols were approved by IVDC with the certificate number IVDC2017-021, and the methods were carried out in accordance with the approved guidelines. To meet experimental ethics requirements, written informed consent for the use of their clinical samples was obtained from the parents of the children whose samples were analysed. All methods were performed in accordance with Polio laboratory manual (World Health Organization, Geneva, Switzerland).

In 1999, 24 stool specimens were collected from twelve AFP patients (2 specimens per person) in support of polio eradication activities in Tibet. World Health Organization requires at least 150 stool samples to be tested every year in order to maintain the detection ability of poliovirus, since there are very few AFP Cases in Tibet every year, stool specimens of healthy children need to be collected to maintain the detection ability. For this reason, 298 stool specimens were collected from 298 healthy children under 5 year old (1 specimen per child) in Tibet in 1999.

In this study, 51 serum samples were random collected for sero-epidemiology study by using serum neutralization tests. Serum samples were obtained from children aged 2.5–5 in Tibet, 39 of which were from Lhasa and 12 from Shigatse, from healthy children showing no signs of disease during the collection process. Serum samples used in this study were collected by the Tibet Center for Disease Control and Prevention, and written informed consent for the use of their clinical samples was obtained from the parents of the children whose samples were analysed. All methods were performed in accordance with Polio laboratory manual (World Health Organization, Geneva, Switzerland).

### Virus isolation and plaque purification

We processed stool samples of AFP patients and healthy children according to standard procedures^38^; then, samples were inoculated into human rhabdomyosarcoma (RD) cells and a mouse cell line with the human poliovirus receptor (L20B). Both cell lines were supplied by the WHO Global Poliovirus Specialized Laboratory in the United States and purchased from the American Type Culture Collection (Manassas, VA, USA). Cells were continuously observed after inoculation for seven days, and the culture was harvested if cells showed a complete EV-like cytopathic effect (CPE). If no CPE appears after 7 days, perform a blind passage and continue examination for a further 7 days.

The harvested culture was diluted with 4 gradients (10^−2^, 10^−3^, 10^−4^, 10^−5^), and the diluted culture was inoculated into 6-well culture plates containing RD cells. Incubate at 35 °C for 2 hours, add agar medium without neutral red solution, and invert at 37 °C for 2 days, then add agar medium with neutral red solution, culture in the dark (35 °C) after staining, and observe for seven days. After the appearance of white spots, appropriate plaques were selected and inoculated into RD cell culture tubes, after CPE appeared, they were passaged twice and cultures were collected.

### Molecular typing

Viral RNA was extracted from cultured cells using the QIAamp Viral RNA Mini Kit (Qiagen, Hilden, Germany). Then one-step RT-PCR Kit (Perfect Real Time, TaKaRa, Dalian) was used to detect RNA of enteroviruses by enterovirus-specific real-time polymerase chain reaction (Real-Time PCR). If the Real-Time PCR result is positive, the partial *VP1* region sequence was obtained by reverse transcription polymerase chain reaction (RT-PCR) amplification using the PrimScript One-Step RT-PCR Kit Ver. 2 (TaKaRa, Dalian, China) with primers E486/E488 (EV-A universal primers), E490/E492 (EV-B universal primers)and E494/E496 (EV-C universal primers)^39^. The amplified products were further purified using the QIAquick PCR Purification Kit (Qiagen, Hilden, Germany). Each strand was sequenced from both directions at least once using the ABI 3130 Genetic Analyser (Applied Biosystems, Foster City, CA, USA). The obtained sequences were identified by the Online Enterovirus Genotyping Tool^16^, and the obtained partial *VP1* sequences were analysed by the Basic Local Alignment Search Tool (BLAST). Sequences were compared with those in the GenBank database to confirm the enterovirus type isolated in Tibet.

### Full-length genome sequencing

The 5′ end of the viral genome sequence was amplified by the 5′-Full RACE Kit (Takara, Shiga, Japan) according to the manufacturer’s instructions, and the 3′ end was amplified using an oligo-dT primer (primer 7500 A)^40^. The *VP4* sequence was amplified by special primers (EVP4 and OL68-1)^41^. The other part of genomic sequences were obtained by designing specific primers using the primer walking method (Table 2). The RT-PCR products were purified using the QIAquick PCR Purification Kit (Qiagen, Hilden, Germany) and sequenced using an ABI 3130 Genetic Analyzer (Applied Biosystems, Foster City, CA, USA).

**Table 2:** RT-PCR and sequencing primers.

Primer	Nucleotide position	Primer sequence(5′-3′)	Orientation	Reference
0001S48		GGGGACAAGTTTGTACAAAAAAGCAGGCTTTAAAACAGCTCTGGGGTT	Forward	^40^
5′RACE-inner-primer-EVB83-996A	996-1015	TGTTGAGTTGCCCAGAGTGA	Reverse	This study
5′RACE-outer-primer-EVB83-1152A	1152-1171	CGTTTTCTCCCAATCCACCG	Reverse	This study
EVP4	541–560	CTACTTTGGGTGTCCGTGTT	Forward	^41^
OL68-1	1178–1197	GGTAAYTTCCACCACCANCC	Reverse	^41^
EVB83-1009S	1009-1028	AACTCCACCATCACCACACA	Forward	This study
EVB83-1857A	1857-1876	CAACCTCCGCGATTTCCATC	Reverse	This study
EVB83-1461S	1461-1480	CTGGGATGGGAGTAGCAGTG	Forward	This study
EVB83-2426A	2426-2445	GGGTCGTTTTGATACAGGGC	Reverse	This study
490	2226–2248	TGIGTIYTITGYRTICCITGGAT	Forward	^39^
491	2883–2902	ATGTAYRTICCICCIGGNGG	Forward	^39^
492	2953–2934	GGRTTIGTIGWYTGCCA	Reverse	^39^
493	3641–3622	TCNACIANICCIGGICCYTC	Reverse	^39^
EVB83-2693S	2693-2712	GGCGCATAAACACGAGAGAG	Forward	This study
EVB83-3142A	3142-3161	GATCACTCTGGGAAGGTGCA	Reverse	This study
EVB83-3309S	3309-3328	ATCAGGGGCTGCTTATGTGG	Forward	This study
EVB83-4084A	4084-4103	GCAATCCACTCCATCCCCTT	Reverse	This study
EVB83-3790S	3790-3810	CACCAACCAGATTTGTGAGCA	Forward	This study
EVB83-4971A	4971-4990	GTGCCTGTGGTTGTATTCCC	Reverse	This study
EVB83-4708S	4708-4727	GCTGGCTCTATCAATGCACC	Forward	This study
EVB83-5947A	5947-5967	AGGTGTGTTGATGACTGGGAA	Reverse	This study
EVB83-4971S	4971-4990	GGGAATACAACCACAGGCAC	Forward	This study
EVB83-5929A	5929-5948	GGAATCCCGCGTCTTTTGAG	Reverse	This study
EVB83-5979S	5979-5998	ACTGGAGCCAAGTGTCTTCC	Forward	This study
EVB83-6773A	6773-6792	ACTAGTGCCAGAACATCCCG	Reverse	This study
EVB83-6541S	6541-6560	TGTGACCCTGACCTCTTCTG	Forward	This study
EVB83-6627S	6627-6646	CTTGAGCCCTGTGTGGTTTG	Forward	This study
7500 A		GGGGACCACTTTGTACAAGAAAGCTGGG(T)^24^	Reverse	^40^

### Bioinformatics analysis

The full-length genomic and amino acid sequences of the six EV-B83 were aligned with those of the EV-B prototype strains using the Muscle algorithm in MEGA7^42–44^. Following alignment, sequences were imported to BioEdit to obtain the identity matrix^45,46^.

EV-B83 sequences longer than 600 nt were obtained from GenBank database, and sequences containing the *VP1* region were screened out. After comparison, some *VP1* coding regions (633 nt in length and located in the EV-B83 prototype strain at nt 2528–3160) were selected to construct phylogenetic trees. For evolutionary analysis, we also selected the sequences of EV-B prototype strains in the GenBank, Yunnan EV-B83, and six Tibet EV-B83 strains to construct the maximum-likelihood phylogenetic trees of *VP1*, *P1*, *P2*, and *P3* coding regions. The sequences were processed by ModelGenerator0.85 to confirm GTR  + G model as the best model, and the maximum-likelihood tree was constructed in MEGA7 with 1000 bootstrap replicates^47^.

Meanwhile, BLAST was used to analyse the *P2* and *P3* sequence regions of the six strains, and full-length sequences with over 85% similarity in GenBank were obtained as potential parents. We used the Recombination Detection Program 4 (RDP4, v4.46) for preliminary recombination analysis and selected RDP, GENECONV, Chimaera, MaxChi, Bootscan, SiScan, and 3Seq for recombination detection^18,48^. For recombinant analysis, we set *P-*values less than 0.01 to obtain more accurate results. We only considered sequences supported by at least three RDP4 methods as potential parental sequences. Since BLAST showed that the *P3* region of some CV-A9 strains had high similarity with the *P3* region of six Tibet EV-B83 strains, we downloaded the full-length genomic sequences of the most similar CV-A9 strains in the GenBank database as potential parents. The CV-B6 and EV-B106 strains (GenBank accession numbers AF039205 and KF990476) in the maximum-likelihood phylogenetic tree of the *P2* and *P3* regions were closest to six Tibet EV-B83 strains and were also used as potential parents. We continuously screened a variety of potential parents using the SimPlot program (200-nt window moving in 20-nt steps) and the maximum-likelihood phylogenetic tree to obtain more reliable potential parental sequences.

### Neutralization titration test

Six Tibet EV-B83 strains were purified by plaque assay, and the titres of them were measured three times, and the average value was taken. The strain with the highest titre, 99258, was selected for the neutralization test. We selected 51 serum samples from children in Tibet for inactivation at 56 °C for 30 minutes, diluted the inactivated serum into five gradients (1:4, 1:16, 1:64, 1:256, 1:1024), and added 50 μL of each serum dilution to a 96-well plate in duplicate. Then, 50 μL of the virus dilution (100 CCID_50_) was added to each well, and the 96-well plate was placed in a 5% CO_2_ incubator at 36 °C. After neutralizing for 2 h, 100 μL RD cell solution was added to each well. Serum control (1:4 serum dilution without virus) and cell control were set up in the experiment. A back titration of challenging virus is included in each test to allow calculation of the titre of virus actually present in that test. After all cells were added, the 96-well plates were placed in a 5% CO_2_ incubator and cultured at 36 °C. After continuous observation for 7 days, the highest serum dilution showing protection from CPE in 50% of cultures was determined. If a neutralizing antibody titre was observed at a serum dilution > 1:8, the serum sample was considered positive and the geometric mean titre (GMT) was calculated. We also used SPSS Statistics software (v19.0) (SPSS Inc., Chicago, IL, USA) for statistical analysis and selected the chi-square test to compare differences in seroprevalence between Lhasa and Shigatse.

### Temperature sensitivity test

In 24-well plates, monolayer RD cells were used to determine the temperature sensitivity of six purified EV-B83 strains, and temperature-sensitive and temperature–resistance strains (*HTPS-QDH11F/XJ/CHN/2011* and *HTYT-ARL-AFP02F/XJ/CHN/2011*, respectively) were selected as control strains^20,26^. Then, 50 μL of undiluted strains were inoculated into 24-well plates and cultured in incubators at optimal and supraoptimal temperatures for viral culture (36 and 39.6 °C, respectively). After 1 h, the unadsorbed virus was removed, and 100 μL maintenance medium was added to each well. The 24-well plates were incubated at 36 or 39.5 °C, and the virus was harvested at 4, 8, 16, 24, and 48 h post-infection. The 50% cell culture infectious dose (CCID_50_) was calculated by the end-point dilution method on monolayer RD cells in 96-well plates at 36 °C. If the titre of the same virus isolate at different temperatures at the same time point were reduced by more than two logarithms, the strain was considered to be temperature-sensitive^49^.

### Nucleotide sequence accession number

The full-length genomic sequences of the six EV-B83 strains (*99245*, *99258*, *99267*, *99268*, *99269*, *99275*) described in this study were uploaded to the GenBank (accession numbers MN164683-MN164688).

## Data Availability

The nucleotide sequences of the entire genome for the six strains determined in this study have been deposited in GenBank nucleotide sequence database under accession numbers MN164683-MN164688.
